# Hordeum vulgare ethanolic extract mitigates high salt-induced cerebellum damage via attenuation of oxidative stress, neuroinflammation, and neurochemical alterations in hypertensive rats

**DOI:** 10.1007/s11011-023-01277-5

**Published:** 2023-08-30

**Authors:** O. A. Ahmed-Farid, Areeg M. Abdelrazek, Hend Elwakel, Maha M. Mohamed

**Affiliations:** 1 Department of Physiology, Egyptian Drug Authority, Giza, 12553 Egypt; 2 Physiology Department Egyptian Drug Authority, Giza, Egypt; 3 Faculty of Medicine, Benha University, Qualubya, Egypt; 4 Home Economic Department, Faculty of Women for Arts Science and Education, Ain Shams University, Asmaa Fahmi, Al Golf, Nasr City, 11757 Cairo Governorate Egypt

**Keywords:** Cerebellum, Oxidative Stress, Inflammation, Neurotransmitters, Barley, Rats

## Abstract

High salt intake increases inflammatory and oxidative stress responses and causes an imbalance of neurotransmitters involved in the pathogenesis of hypertension that is related to the onset of cerebral injury. Using natural compounds that target oxidative stress and neuroinflammation pathways remains a promising approach for treating neurological diseases. Barley (Hordeum *vulgare L*.) seeds are rich in protein, fiber, minerals, and phenolic compounds, that exhibit potent neuroprotective effects in various neurodegenerative diseases. Therefore, this work aimed to investigate the efficacy of barley ethanolic extract against a high salt diet (HSD)-induced cerebellum injury in hypertensive rats. Forty-eight Wistar rats were divided into six groups. Group (I) was the control. The second group, the HSD group, was fed a diet containing 8% NaCl. Groups II and III were fed an HSD and simultaneously treated with either amlodipine (1 mg /kg b.wt *p.o*) or barley extract (1000 mg /kg b.wt *p.o*) for five weeks. Groups IV and V were fed HSD for five weeks, then administered with either amlodipine or barley extract for another five weeks. The results revealed that barley treatment significantly reduced blood pressure and effectively reduced oxidative stress and inflammation in rat's cerebellum as indicated by higher GSH and nitric oxide levels and lower malondialdehyde, TNF-α, and IL-1ß levels. Additionally, barley restored the balance of neurotransmitters and improved cellular energy performance in the cerebellum of HSD-fed rats. These findings suggest that barley supplementation exerted protective effects against high salt-induced hypertension by an antioxidant, anti-inflammatory, and vasodilating effects and restoring neurochemical alterations.

**Graphical abstract:**

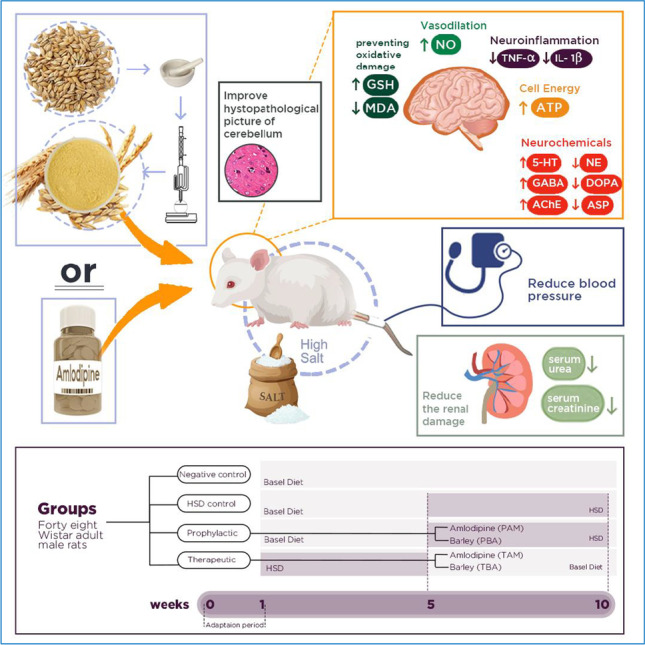

## Introduction

Hypertension is a public health problem, affecting roughly 1.5 billion people, and accounts for millions of fatalities annually (Qin et al. 2021). This complex disease is a significant risk factor for ischemic brain infarction, intracranial hemorrhage, myocardial infarction, congestive heart failure, and kidney damage (Fedoce et al. 2018; Borrelli et al. 2020; Qin et al. 2021). Epidemiological and interventional research has shown that the interactions between multiple genetic and environmental factors may result in the development of high blood pressure (Waken et al. 2017; Fedoce et al. 2018). Dietary choices, particularly salt intake, are a significant environmental factor strongly associated with increased blood pressure and the aggravation of hypertension (Olde Engberink et al. 2017; Grillo et al. 2019; Borrelli et al. 2020). Furthermore, high-salt diets have been linked to cerebrovascular diseases and cognitive impairment in humans (Fedoce et al. 2018) and rodents (Zhang et al. 2020; Du et al. 2021; Leal et al. 2022).

Many studies have established that the hyperactive sympathetic nerve is strongly associated with the initiation and progression of hypertension (Zhang et al. 2020; Du et al. 2021). Many excitatory and inhibitory neurotransmitters influence sympathetic neural activity. Glutamate and norepinephrine (NE) (a marker of sympathetic activity) are excitatory neurotransmitters, and gamma-aminobutyric acid (GABA) is a dominant inhibitory neurotransmitter, which is known to be involved in inducing sympathetic response (Gao et al. 2017). The hypertensive responses are due to increased excitatory adrenergic and glutamatergic activities and a decrease in GABAergic activity in the hypothalamic paraventricular nucleus (PVN) (Gao et al. 2017; Du et al. 2021). There are accumulating evidence suggesting that salt-induced hypertension leads to an imbalance between neurotransmitters, decreased inhibitory neurotransmitter GABA and increased excitatory adrenergic and glutamatergic neurotransmitters (Gao et al. 2017; Du et al. 2021; Leal et al. 2022). Furthermore, a large body of evidence indicates that oxidative stress in the brain plays an essential role in the pathogenesis of hypertension. Many studies have found that a high salt diet produces an excessive amount of reactive oxygen species (ROS) that is essential in modulating blood pressure and sympathetic nerve activity in hypertension (Gao et al. 2017; Yu et al. 2021). Additionally, substantial evidence demonstrates that high salt intake increased pro-inflammatory cytokines, such as tumor necrosis factor-alpha (TNF-α), interleukin-1β (IL-1β), and the IL-6, decreased anti-inflammatory cytokines, such as IL-10, in the PVN (Qi et al. 2016; Knoll et al. 2017; Bhusal et al. 2021). Thus, restoring the balance between the excitatory and inhibitory neurotransmitters and reducing the production of ROS and pro-inflammatory cytokines in the brain is beneficial in treating hypertension.

Barley (*Hordeum vulgare* L., *H. vulgar*), a member of the Poaceae family, has been one of the most significant food grains since ancient times (Idehen et al. 2017). Owing to its nutritional and bioactive compounds, barley is highly desired for its health benefits. Its flour is reported to contain 54.2% of starch, 2.42% of crude fat, 2.92% of total ash, 14.2% of protein, 2.86% of soluble dietary fiber, 10.24% of insoluble dietary fiber, and 13.1% of total dietary fiber (Huang et al. 2020). Barley includes a variety of functional ingredients, especially β-glucan, phenolic acids, flavonoids, phytosterols, alkyl resorcinols, benzoxazinoids, lignans, and folate. These bioactive compounds contribute to its various health benefits, such as anti-inflammatory and antioxidative activity against different free radicals. Additionally, barley is a good source of valuable substances, including vitamins B1, B2, and B3, and vitamin E, which are crucial for maintaining the neurological system (Zeng et al. 2020). Besides, barley proteins have been recognized as an abundant source of essential amino acids such as lysine, threonine, methionine, tryptophan, tyrosine, and phenylalanine (Jaeger et al. 2021). As tryptophan is a precursor for serotonin and tyrosine and phenylalanine are precursors of catecholamines (Fernstrom and Fernstrom 2007; Badrasawi et al. 2013) thus, barley extract may play a role in restoring any probable imbalance between the excitatory and inhibitory neurotransmitters that could be caused by high salt diet.

Amlodipine, calcium channel blocker, is one of the most extensively antihypertensive drugs, and was effective for protecting stroke and myocardial infarction (Kim et al. 2022). Amlodipine has also been noted to have several additional effects, including improvement of coronary microvascular endothelial dysfunction, and an increase in the production of NO in vascular endothelial cells, as well as antioxidant actions (Mawatari et al. 2007).

For the reasons mentioned above, the current study was carried out to compare the prophylactic and therapeutic efficacy of the barely ethanolic extract against cerebellum damage in high-salt-induced hypertensive rats, focusing on restoring the balance between the excitatory and inhibitory neurotransmitters and attenuating oxidative stress and inflammation in the cerebellum of rats. The study also aimed to compare the efficacy of barley extract treatments to amlodipine, the widely used antihypertensive drug, treatments.

## Materials and methods

### Chemicals

Amlodipine besylate, reduced glutathione reference standard, adenosine triphosphate (ATP), and 1,1,3,3- Tetraethoxypropane were purchased from Sigma Aldrich (St. Louis, MO, USA), while perchloric acid and methanol (HPLC grade) were obtained from Loba Co. (India). 5,5'-Dithio-bis-[2-nitrobenzoic acid] (DTNB), nicotinamide adenine dinucleotide phosphate (NADP), sulphosalsilic Acid, P-amino benzyl glutamate, and acetylthiocholine iodide were purchased from TM MEDIA Co. (India). Potassium dichromate, monobasic potassium phosphate, nitrites, and nitrate were from Al Nasr chemicals (Abozabal- Qualybia- Egypt). All chemicals were analytical grade.

### Experimental animals

Forty-eight Wistar adult male rats (180–200 g) were used for this study. The animals were obtained from the National Organization for Drug Control and Research animal house, NODACR, Giza, Egypt. The rats were housed individually in wire mesh cages under standard conditions (temperature 25—28° C, 12h light and 12h darkness cycles) with free access to basal diet (AIN-93) and water (Reeves et al. 1993). All animal procedures in the present study were approved by the Animal Ethics Committee of Women’s College, Ain Shams University (approval no. ASU/W/Sci-7R/23–2-17).

### Extract preparation

Barley grains were obtained from the Field Crops Research Institute, Agriculture Research Centre, Giza, Egypt. The grains were cleaned and grounded into a fine powder. To obtain the ethanolic extract, 70% aqueous ethanol solution was added to the powdered barley samples at a ratio of 20:1 (w/w) and were extracted twice using a two h reflux extraction. The extract was concentrated under reduced pressure. The concentrate was filtered, lyophilized, and subsequently stored at 4^∘^C.

### Experimental design

After the acclimatization period (one week), rats were randomly assigned into six groups (eight rats/group). Group (1): were maintained on a basal diet for ten weeks (negative control group). Group (II): were maintained on a basal diet containing 8% NaCl for five weeks [high salt diet (HSD), high salt control]. Group (III): were maintained on a diet containing 8% NaCl and parallel administered with amlodipine (1 mg /kg body weight, *p.o*) (Lee et al. 2021) for five weeks (prophylactic amlodipine, HSD + PAM)). Group (IV) was maintained on a diet containing 8% NaCl and parallel administered with barley ethanolic extract (1g /kg body weight *p.o*) (Darwish et al. 2013) for five weeks (prophylactic barley, HSD + PBA). Group (V) nourished 8% salt in the diet for five weeks, then administered with amlodipine 1 mg /kg body weight *p.o* for another five weeks (therapeutic amlodipine, HSD + TAM). Group (VI) nourished 8% salt in the diet for five weeks, then administered with barley extract 1000 mg /kg body weight *p.o* for another five weeks (therapeutic barley, HSD + TBA).

### Measurement of systolic and diastolic blood pressure

Systolic and diastolic blood pressure of conscious rats was measured noninvasively via a tail-cuff instrument as described previously (Walkowska et al. 2015).

### Blood collection and tissue preparation

At the end of the experiment, animals from each group were sacrificed by decapitation, and blood samples were collected from the retro-orbital plexus veins. Serum was separated by centrifugation at 3000 r.p.m. for 20 min and kept at -20°C until further biochemical analysis. Cerebellum samples were taken at the time of sacrifice. The tissue was immediately excised and homogenized using a homogenizer surrounded with an ice jacket and with 10% potassium chloride in a dilution of 1:10 of tissue homogenate, followed by centrifugation in a cooling centrifuge at 4°C for 20 min at 5000 r.p.m. The homogenates were used to determine GSH, MDA, NOx, TNF-α, IL-1ß, NE, DA, 5HT, GABA, ASP, ATP, and AChE.

### Biochemical analysis

#### Determination of serum urea and creatinine

Urea and creatinine serum levels were assessed using Biodiagnostic kits (Randox Laboratories, Crumlin, U.K.) by modifying methods based on diacetylmonoxime reaction (Marsh et al. 1965) and Jaffe’s reaction (Biod and Sirota 1948).

#### Determination of cerebellum levels of reduced glutathione, malondialdehyde, nitric oxide, monoamines, amino acids, and adenosine triphosphate

For measurement of cerebellum levels of reduced glutathione (GSH), malondialdehyde (MDA), nitrites and nitrate (NOx), norepinephrine (NE), dopamine (DA) and 5-hydroxytryptamine (5-HT), gamma-aminobutyric acid (GABA) and aspartic acid (ASP), HPLC system (Agilent HP 1200 series, USA) consisting of a quaternary pump, column oven, Rheodine injector and 20 µl loop, and UV variable wavelength detector was used. The report and chromatograms were taken from the Chemstation program (Agilent, USA).

For detection of the thiol compound of GSH, 30 cm × 3.9 mm C-18 μ Bondapak column was used. The flow rate was 1ml/min, and UV detection at wavelength 190 nm was applied. 25 mmol sodium phosphate buffer, pH 3.5, containing five mmol tetrabutylammonium phosphate and 13% methanol, was used as the mobile phase. Samples were compared to the reduced glutathione reference standard purchased from Sigma Chemical Co. The results were expressed as μmol/g tissue (Jayatilleke and Shaw 1993). For determination of MDA level, the samples The analytical column was Supelcosil C18 (particle size: 5 µm, pore size: 80 A^o^) (250 × 4.5 mm ID). The mobile phase was 82.5:17.5 (v/v) 30 mM monobasic potassium phosphate (pH 3.6), and methanol of HPLC grade, with a flow rate of 1.2 ml/min, wavelength 250 nm, was applied for detection. For preparing the MDA standard, 25 μl 1,1,3,3 tetra-ethoxy-propane (TEP) was dissolved in 100 ml water to produce a one mM stock solution. The working standard was prepared by hydrolyzing 1 ml of TEP stock solution in 50 ml 1% sulfuric acid, followed by 2 h of incubation at room temperature. The resultant MDA standard of 20 nmol/ml was further diluted with 1% sulfuric acid to produce the final concentration of 1.25 nmol/ml, which served as the standard for estimating total MDA (Karatepe 2004). Nitric oxide was determined as the ratio of nitrites and nitrate (NOx) by HPLC (Papadoyannis et al. 1999). The analytical column was anion exchange PRP-X100 Hamilton, 150 × 4.1 mm, ten μm. The mobile phase was a mixture of 0.1 M NaCl—methanol at a volume ratio of 45:55. The flow rate of 2 mL/min, and the wavelength was adjusted to 230 nm. A standard mixture of nitrite and nitrate was used to determine the retention times and separation of the peaks.

For monoamines determination, the samples were immediately extracted from the trace elements and lipids using solid phase extraction CHROMABOND column (NH2 phase cat. No. 730031). The samples were injected directly into an Aqua 5μm 18 200 Ao, LC Column 150 × 4.6 mm, purchased from Phenomenex, USA, under the following conditions: mobile phase 20 mM potassium phosphate, pH 2.7, flow rate 1.5 ml/min, UV wavelength 290 nm (Pagel et al. 2000). Brain GABA and Asp were detected by HPLC using the precolumn phenylisothiocyanate (PITC) derivatization technique (Heinrikson and Meredith 1984). The chromatographic system and the column for measuring GABA and Asp were of the same composition as described above.

ATP content was determined by injection of samples into a system consisting of an analytical column Nucleosil C-18 (15 × 0.4 cm). The mobile phase for the adenine nucleotides was 50 mM potassium phosphate 1% (v/v) methanol at PH 5.5 and a flow rate of 1 ml/ min. The UV detector was set at 210 nm (Teerlink et al. 1993).

#### Determination of tissue TNF-α and IL-1ß (pg / mL)

The concentration of cytokines (TNF-α and IL-1ß) was determined in the cerebellum using commercially available ELISA assays, following the instructions supplied by the manufacturer (DuoSet kits, R&D Systems; Minneapolis). The results are shown as pg of cytokine /mL.

#### Determination of Acetylcholinesterase activity (μmol SH/min/g tissue)

The procedure used for the determination of acetylcholinesterase activity (AChE) in the brain cerebellum samples of rats is a modification of the Ellman et al. (1961) method as described by Gorun et al. (1978). AChE activity was determined after extraction using the following protocol: 0.14-ml phosphate buffer 20 mmol (pH 7.6), 0.05 ml of 5-mmol-acetylthiocholine iodide, and 0.01 ml of tissue homogenate were pipetted in a cuvette. After 10 min of incubation at 38 °C, the reaction was stopped with 1.8 ml of DTNB – phosphate ethanol reagent. The color was read immediately at 412 nm using a Shimadzu spectrophotometer UV –1601. The cholinesterase activity was determined as µmol SH from a standard curve.

### Histological assays

The rat brains were carefully excised and fixed in Bouin’s fixative for histological study. After fixation, the brains were processed in ascending grades of ethyl alcohol, cleared in xylol, and implanted in paraffin wax at 60°C. Serialized 5–6 μm sections were cut by a Cambridge Rocking Microtome (Cat. No. 52111, London) and affixed on slides. Hematoxylin and eosin staining was applied to the sections on the slides (Drury and Wallington 1980).

### Statistical analysis

Data were statistically analyzed using SPSS 18.0 software (SPSS, Chicago, IL, USA) and are expressed as the mean ± SEM. One-way analysis of variance was used, followed by Post hoc test (Bonferroni test) for comparison among multiple groups.

## Results

### Effects of barley and amlodipine on systolic and diastolic blood pressure levels in salt-induced hypertensive rats

**Fig. 1:**
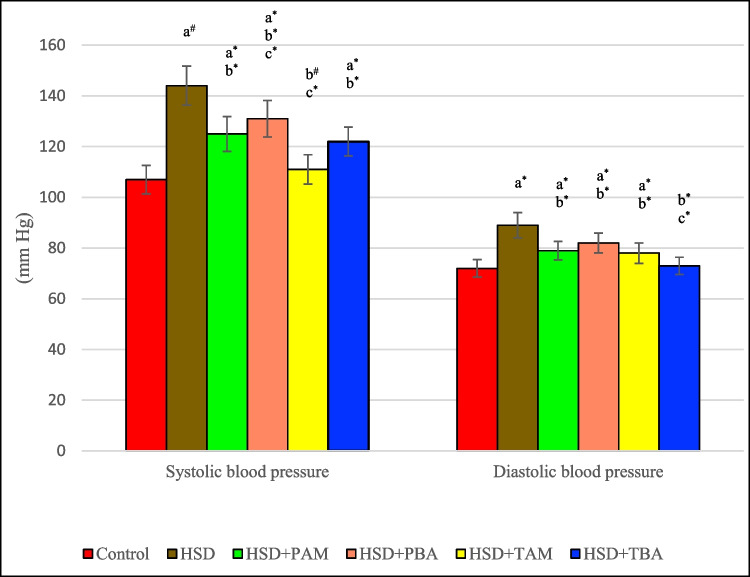
Systolic and diastolic blood pressure of rats on the normal salt diet (negative control), or 8% salt diet (HSD group), HSD + amlodipine for five weeks (HSD + PAM), HSD and barley extract for five weeks (HSD + PBA), HSD for five weeks then amlodipine for another five weeks (HSD + TAM) or HSD for five weeks then barley extract for another five weeks (HSD + TBA). Data are presented as mean values ± SEM; (*n* = 8). a means a significant difference from the control negative group, b means a significant difference from HSD group, and c means a significant difference from HSD + PAM group. *, # and † represent statistical significance at *p* < 0.05, *p* < 0.01 and *p* < 0.001, respectively (one-way ANOVA followed by Post hoc test)

Figure 1 demonstrate that rats on HSD displayed a significant increase in systolic (34.5%, *p* < 0.01) and diastolic blood pressure (23.6%, *p* < 0.05) levels compared to negative control. On the other hand, prophylactic and therapeutic treatment with amlodipine and barley extract mitigated high salt-induced hypertension compared with the HSD group. However, prophylactic and therapeutic amlodipine treatments are more effective in reducing blood pressure than barley treatment (Table 1).

**Table 1:** Effects of amlodipine and barley extracts on the serum levels of urea and creatininein salt-induced hypertensive rats

Groups		Parameters	
		Urea	Creatinine
I	Control	29.90 ± 0.842	0.751 ± 0.021
II	HSD	41.26 ± 1.151^a#^	1.298 ± 0.037a ^†^
III	HSD + PAM	40.36 ± 1.15^a#^	1.268 ± 0.036a ^†^
IV	HSD + PBA	36.57 ± 1.04^a*b*c*^	0.926 ± 0.026^a*b*C^^†^
V	HSD + TAM	37.32 ± 1.057^a*b*^	1.064 ± 0.029^a*b*^
VI	HSD + TBA	36.25 ± 0.968^a*b*c*^	1.091 ± 0.030^a*b*^

Data are expressed as Mean ± SEM for 8 rats /group

a significant difference from the control group in the same column

b significant difference from HSD in the same column

c significant difference from HSD + PAM in the same column

^*^, # and † represent statistical significance at *p* < 0.05, *p* < 0.01 and *p* < 0.001, respectively (one-way ANOVA followed by Post hoc test)

### Effects of prophylactic and therapeutic treatment with amlodipine and barley extract on serum urea and creatinine in salt-induced hypertensive rats

**Fig. 2:**
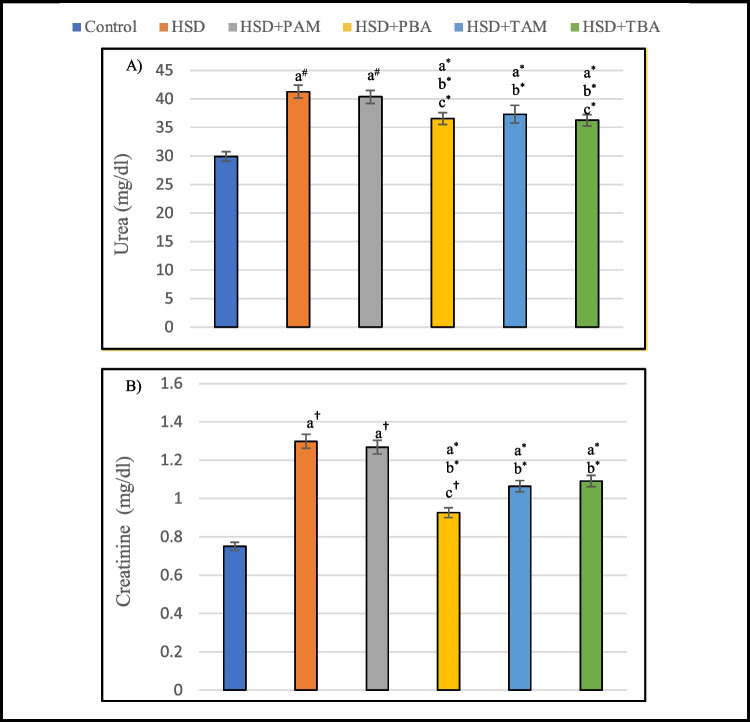
Serum levels of urea (**A**) and creatinine (**B**) of rats on the normal salt diet (negative control), or 8% salt diet (HSD group), HSD + amlodipine for five weeks (HSD + PAM), HSD and barley extract for five weeks (HSD + PBA), HSD for five weeks then amlodipine for another five weeks (HSD + TAM) or HSD for five weeks then barley extract for another five weeks (HSD + TBA). Data are presented as mean values ± SEM; (*n* = 8). a means a significant difference from the negative control group, b means a significant difference from the HSD group, and c means a significant difference from the HSD + PAM group. *, # and † represent statistical significance at *p* < 0.05, *p* < 0.01 and *p* < 0.001, respectively (one-way ANOVA followed by Post hoc test)

As shown in Fig. 2a and b, HSD caused kidney function deterioration as indicated by a significant elevation in serum urea (37.9%, *p* < 0.01) and creatinine (72.8%, *p* < 0.001) levels compared with the negative control group. However, barley extract administration (prophylactic and therapeutic treatment) significantly (*P* < 0.05) reduced this rise in serum urea (11.3% and 12.1%) and creatinine (28.6% and 15.9%) levels as compared to the HSD group. On the other hand, prophylactic amlodipine treatment had no significant effects (*P* > 0.05) on serum urea and creatinine. While therapeutic amlodipine treatment significantly (*P* < 0.05) reduced their levels by 9.5% and 18%, respectively, as compared to the HSD group.

### Effects of prophylactic and therapeutic treatment with amlodipine and barley extract on oxidative stress, nitric oxide, and inflammatory cytokines in salt-induced hypertensive rats

**Fig. 3:**
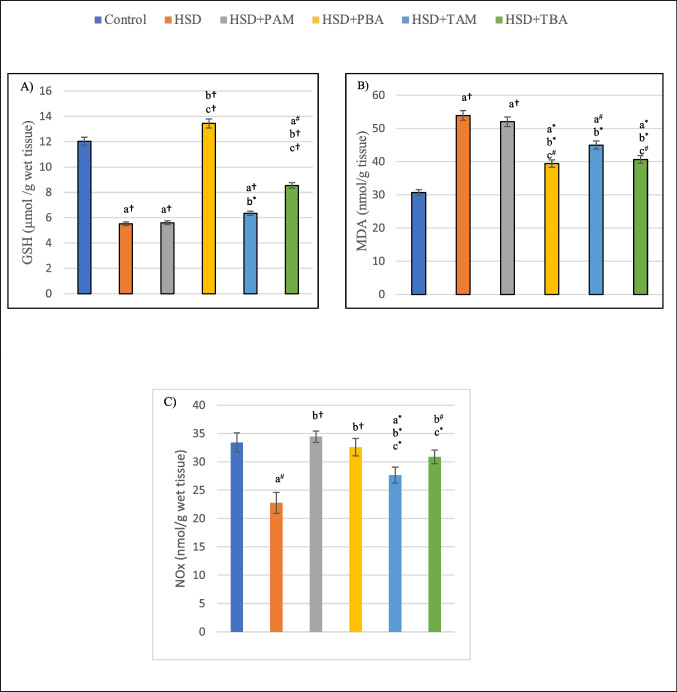
Cerebral levels of (**A**) reduced glutathione (GSH), (**B**) malondialdehyde (MDA), and (**C**) nitric oxide (NOx) of rats on the normal salt diet (negative control), or 8% salt diet (HSD group), HSD + amlodipine for five weeks (HSD + PAM), HSD and barley extract for five weeks (HSD + PBA), HSD for five weeks then amlodipine for another five weeks (HSD + TAM) or HSD for five weeks then barley extract for another five weeks (HSD + TBA). Data are presented as mean values ± SEM; (*n* = 8). a means a significant difference from the negative control group, b means a significant difference from the HSD group, and c means a significant difference from the HSD + PAM group. *, # and † represent statistical significance at *p* < 0.05, *p* < 0.01 and *p* < 0.001, respectively (one-way ANOVA followed by Post hoc test)

**Fig. 4:**
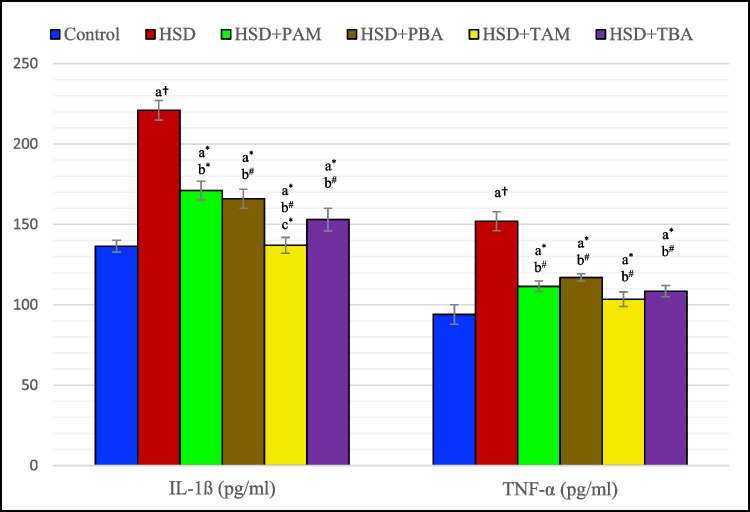
Cerebral levels of interleukin-1 ß (IL- ß) and tumor necrosis factor- α (TNF- α) in rats on the normal salt diet (negative control), or 8% salt diet (HSD group), HSD + amlodipine for five weeks (HSD + PAM), HSD and barley extract for five weeks (HSD + PBA), HSD for five weeks then amlodipine for another five weeks (HSD + TAM) or HSD for five weeks then barley extract for another five weeks (HSD + TBA). Data are presented as mean values ± SEM; (*n* = 8). a means a significant difference from the negative control group, b means a significant difference from the HSD group, and c means a significant difference from HSD + PAM group. *, # and † represent statistical significance at *p* < 0.05, *p* < 0.01 and *p* < 0.001, respectively (one-way ANOVA followed by Post hoc test)

Data demonstrated by Fig. 3 show that GSH and NOx levels were significantly reduced by (49.9%, *p* < 0.001 and 31.9%, *p* < 0.01 respectively) while MDA level was significantly (*P* < 0.001) elevated by (75.6%) in the cerebellum of rats fed on HSD as compared to negative control. In addition, levels of TNF-α and IL-1ß were significantly elevated (*P* < 0.001) by 61.7% and 62.5%, respectively, in the cerebellum of HSD-fed rats as compared to negative control (Fig. 4). The oxidative stress, inflammation, and reduction in NOx level induced by feeding HSD were significantly ameliorated in barley extract-treated groups. Both prophylactic and therapeutic barley treatments increased the levels of GSH (143% and 54.86%, *P* < 0.001) and NOx (43.23%, *P* < 0.001 and 35.7%, *p* < 0.01) and decreased the levels of MDA (26.8% and 24.63%, *p* < 0.05), TNF-α (23% and 33.5%, *P* < 0.01) and IL-1ß (24.8% and 34.3%, *P* < 0.01) as compared to HSD groups. Similarly, therapeutic amlodipine treatment significantly increased the levels of GSH (15%, *P* < 0.05) and NOx (21.5%, *P* < 0.01) and decreased the levels of MDA (16.5%, *P* < 0.05), TNF-α (31.9%, *P* < 0.01), and IL-1ß (37.1%, *P* < 0.01). On the other hand, the PAM + HSD group showed no significant changes (*P* > 0.05) in the levels of GSH and MDA. In contrast, it showed a significant (*P* < 0.05) increase in the level of NOx by 51.4% (*p* < 0.001) and a significant decrease in the levels of cerebral TNF-α and IL-1 ß (26.6%, *p* < 0.01 and 22.6%, *p* < 0.05) as compared to the HSD group (Table 2, Figs. 3 and 4).

**Table 2:** Effects of amlodipine and barley extracts on the levels of reduced glutathione (GSH), malondialdehyde (MDA), tumor necrosis factor-α (TNF- α), interlukine-1ß (IL-1ß), and nitric oxide (NO) in cerebellum of salt-induced hypertensive rats

Groups		Parameters				
		GSH (µmol /g wet tissue)	MDA (nmol/g wet tissue)	TNF-α (pg/ml)	IL-1ß (pg/ml)	NO (nmol/g wet tissue)
I	Control	11.037 ± 0.897	30.689 ± 0.860	94 ± 6.5	136 ± 10.1	33.427 ± 0.897
II	HSD	5.519 ± 0.149^a†^	53.904 ± 1.483^a†^	152 ± 9.32^a†^	221 ± 17.7^a†^	22.755 ± 1.701^a#^
III	HSD + PAM	5.604 ± 0.151^a†^	52.015 ± 1.233^a†^	111.5 ± 8.69^a*b#^	171 ± 12.6^a*b*^	34.456 ± 1.826^b †^
IV	HSD + PBA	13.44 ± 0.995^b†c†^	39.413 ± 1.095^a*b*c#^	117 ± 9.13^a*b#^	166 ± 11.5^a*b#^	32.593 ± 0.995^b †^
V	HSD + TAM	6.352 ± 0.17^a†b*^	44.969 ± 1.233^a#b*^	103.5 ± 8.79^a*b#^	139 ± 8.97^a*b#c*^	27.65 ± 1.537^a*b*c*^
VI	HSD + TBA	8.54 ± 0.266^a#b† c†^	40.624 ± 1.152^a*b*c#^	101.5 ± 9.1^a*b#^	145 ± 10.1^a*b#^	30.879 ± 2.103^b#c*^

Data are expressed as Mean ± SEM for 8 rats /group

a significant difference from the control group in the same column

b significant difference from HSD in the same column

c significant difference from HSD + PAM in the same column

^*^, # and † represent statistical significance at *p* < 0.05, *p* < 0.01 and *p* < 0.001, respectively (one-way ANOVA followed by Post hoc test)

### Effects of prophylactic and therapeutic treatment with amlodipine and barley extract on the cerebral levels of neurotransmitters and activity of acetylcholinesterase (AChE) in high salt-induced hypertensive rats

**Fig. 5:**
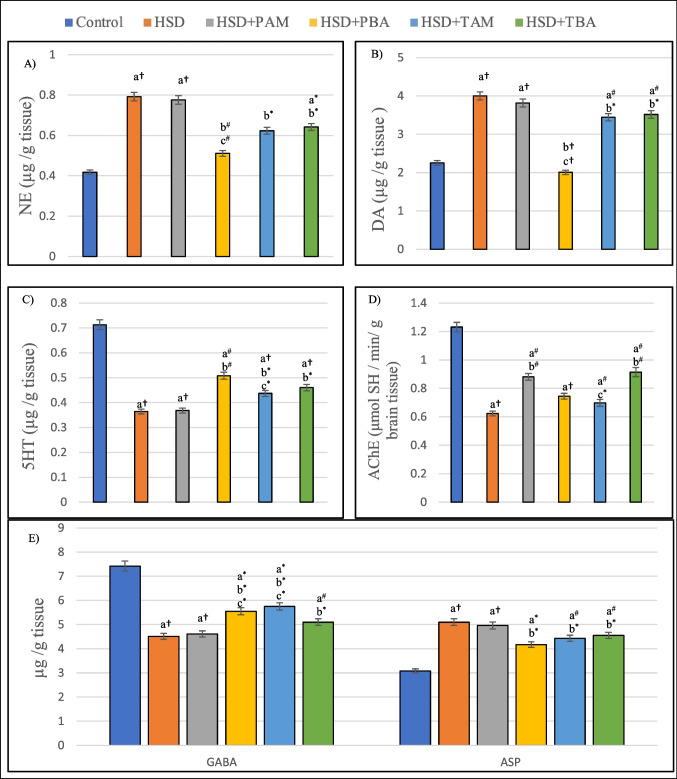
Cerebral levels of (**A**) norepinephrine (NE), (**B**) dopamine (DA), (**C**) 5-hydroxytryptamine (5-HT), (**D**) acetylcholinesterase activity, and (**E**) gamma-aminobutyric acid (GABA) and aspartate (ASP) of rats on normal salt diet (negative control), or 8% salt diet (HSD group), HSD + amlodipine for five weeks (HSD + PAM), HSD and barley extract for five weeks (HSD + PBA), HSD for five weeks then amlodipine for another five weeks (HSD + TAM) or HSD for five weeks then barley extract for another five weeks (HSD + TBA). Data are presented as mean values ± SEM; (*n* = 8). a means a significant difference from negative control group, b means asignificant difference from HSD group, and c means significant difference from HSD + PAM group. *, # and † represent statistical significance at *p* < 0.05, *p* < 0.01 and *p* < 0.001, respectively (one-way ANOVA followed by Post hoc test)

Data illustrated in Fig. 5  and Table 3 revealed that rats maintained on HSD had significant (*P* < 0.001) higher cerebral levels of NE (89.14%), DA (77.6%), and aspartate (65.58%) and significant (*P* < 0.001) lower levels of 5-HT (48.9%) and GABA (39.2%) as compared to the negative control group. Oral administration of barley (prophylactic and therapeutic) to HSD-fed rats significantly (*P* < 0.05) reduced the levels of NE (35.4%, *p* < 0.01 and 18.9%, *p* < 0.05), DA (49.8%, *p* < 0.001 and 12.2%, *p* < 0.05), and aspartate (18.2% and 10.7%, *p* < 0.05) and augmented the levels of 5-HT (40.1%, *p* < 0.01 and 26.3%, *p* < 0.05) and GABA (23% and 13.08%, *p* < 0.05). Data clarified in Fig. 5D also showed that the activity of acetylcholinesterase (AChE) in the cerebellum of rats maintained in HSD was significantly (*P* < 0.05) reduced by 49.3% as compared to normal rats. Prophylactic amlodipine and therapeutic barley treatments significantly (*P* < 0.01) increased the AChE activity by 41.1% and 46.47%, respectively, as compared to HSD group. On the other hand, HSD + PBA and HSD + TAM groups showed non-significant increase (*p* > 0.05) in cerebral AChE as compared to HSD group (Table 4).

**Table 3:** Effects of barley extract and amlodipine on the levels of norepinephrine (NE), dopamine (DA), 5-hydroxytryptamine (5-HT), gamma-aminobutyric acid (GABA), and aspartate (ASP) in the cerebellum of high salt-induced hypertensive rats (µg /g tissue)

Groups		Parameters (µg /g tissue)				
		NE	DA	5-HT	GABA	ASP
I	Control	0.418 ± 0.011	2.253 ± 0.064	0.713 ± 0.02	7.42 ± 0.21	3.08 ± 0.083
II	HSD	0.792 ± 0.02^a†^	4.01 ± 0.11^a†^	0.364 ± 0.01^a†^	4.51 ± 0.13^a†^	5.10 ± 0.143^a†^
III	HSD + PAM	0.776 ± 0.021^a†^	3.82 ± 0.11^a†^	0.37 ± 0.01^a†^	4.61 ± 0.13^a†^	4.96 ± 0.141^a†^
IV	HSD + PBA	0.511 ± 0.014^b#c#^	2.01 ± 0.06^b†c†^	0.51 ± 0.014^a#b#^	5.55 ± 0.15^a*b*c*^	4.17 ± 0.116^a*b*^
V	HSD + TAM	0.623 ± 0.017^b*^	3.45 ± 0.09^a#b*^	0.44 ± 0.012^a†b*c*^	5.75 ± 0.15^a*b*c*^	4.43 ± 0.125^a#b*^
VI	HSD + TBA	0.642 ± 0.017^a*b*^	3.52 ± 0.1^a#b*^	0.46 ± 0.013^a†b*^	5.1 ± 0.14^a#b*^	4.55 ± 0.127^a#b*^

Data are expressed as Mean ± SEM for 8 rats /group

a significant difference from the control group in the same column

b significant difference from HSD in the same column

c significant difference from HSD + PAM in the same column

^*^, # and † represent statistical significance at *p* < 0.05, *p* < 0.01 and *p* < 0.001, respectively (one-way ANOVA followed by Post hoc test)

**Table 4:** Effects of barley extract and amlodipine on the levels of cell energy and activity of AChE in the cerebellum of high salt-induced hypertensive rats (µg /g tissue)

Groups		Parameters	
		ATP	AChE
I	Control	18.638 ± 0.519	1.231 ± 0.033
II	HSD	10.664 ± 0.295^a†^	0.624 ± 0.017^a†^
III	HSD + PAM	11.086 ± 0.299^a†^	0.881 ± 0.024^a#b#^
IV	HSD + PBA	22.484 ± 0.605^b†c†^	0.745 ± 0.02^a†^
V	HSD + TAM	13.658 ± 0.361^a#b*^	0.692 ± 0.019^a#c*^
VI	HSD + TBA	16.325 ± 0.437^a*b†c*^	0.914 ± 0.025^a#b#^

Data are expressed as Mean ± SEM for 8 rats /group

a significant difference from the control group in the same column

b significant difference from HSD in the same column

c significant difference from HSD + PAM in the same column

^*^, # and † represent statistical significance at *p* < 0.05, *p* < 0.01 and *p* < 0.001, respectively (one-way ANOVA followed by Post hoc test)

### Effects of prophylactic and therapeutic treatment with amlodipine and barley extract on brain cell energy in high salt-induced hypertensive rats

**Fig. 6:**
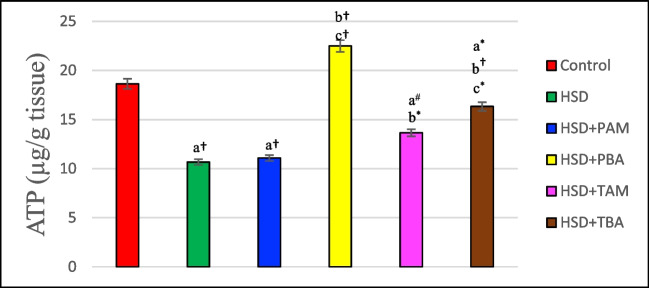
ATP level in the brain of rats on the normal salt diet (negative control), or 8% salt diet (HSD group), HSD + amlodipine for five weeks (HSD + PAM), HSD and barley extract for five weeks (HSD + PBA), HSD for five weeks then amlodipine for another five weeks (HSD + TAM) or HSD for five weeks then barley extract for another five weeks (HSD + TBA). Data are presented as mean values ± SEM; (*n* = 8). a means a significant difference from the negative control group, b means a significant difference from HSD group, and c means a significant difference from HSD + PAM group. *, # and † represent statistical significance at *p* < 0.05, *p* < 0.01 and *p* < 0.001, respectively (one-way ANOVA followed by Post hoc test)

The results presented in Fig. 6 revealed that ATP level was significantly (*P* < 0.001) reduced by 42.78% in the rats maintained on HSD as compared to the negative control. Parallel and therapeutic administration of barley (110% and 53%, *p* < 0.001) to hypertensive rats showed significant improvement and increase in cerebral ATP as compared to HSD group. Meanwhile, only therapeutic amlodipine significantly (*p* < 0.05) increased the level of ATP. In contrast, parallel amlodipine had no significant (*P* > 0.05) effect on ATP level in rats' cerebellum compared to the HSD group (Table 4).

Data presented in Table 5 show the correlation coefficient between parameters indifferent groups of rats.

**Table 5:** Correlation coefficient between parameters in different groups of rats (*p* < 0.01)

	MDA	GSH	NO	TNF-α	IL-1ß	NE	DA	5HT	GABA	ASP	AChE	ATP
MDA	1											
GSH	–0.778632	1										
NO	–0.81325	0.9424734	1									
TNF-α	0.69413353	–0.5390868	–0.5309646	1								
IL-1ß	0.67327442	–0.670902	–0.6145788	0.91546082	1							
NE	0.95307323	–0.8333442	–0.8701812	0.62237257	0.64827141	1						
DA	0.82698601	–0.8925755	–0.9383225	0.4963446	0.55806937	0.92433474	1					
5HT	–0.9211908	0.66337224	0.75718397	–0.533266	–0.4895947	–0.9122707	–0.7873504	1				
GABA	–0.8622848	0.57069084	0.64539202	–0.5635763	–0.5290167	–0.8805652	–0.7146599	0.95760449	1			
ASP	0.94477993	–0.7074475	–0.7718023	0.60758875	0.5611891	0.94672853	0.8291604	–0.953139	–0.9258115	1		
AChE	–0.6847801	0.34566007	0.47439338	–0.6241111	–0.4503758	–0.5623959	–0.4216091	0.76638845	0.7034686	–0.6978842	1	
ATP	–0.7961276	0.93648709	0.94054117	–0.5153208	–0.631147	–0.8476013	–0.9197527	0.68227432	0.56851756	–0.7015485	0.3634374	1

### Histopathological results

**Fig. 7:**
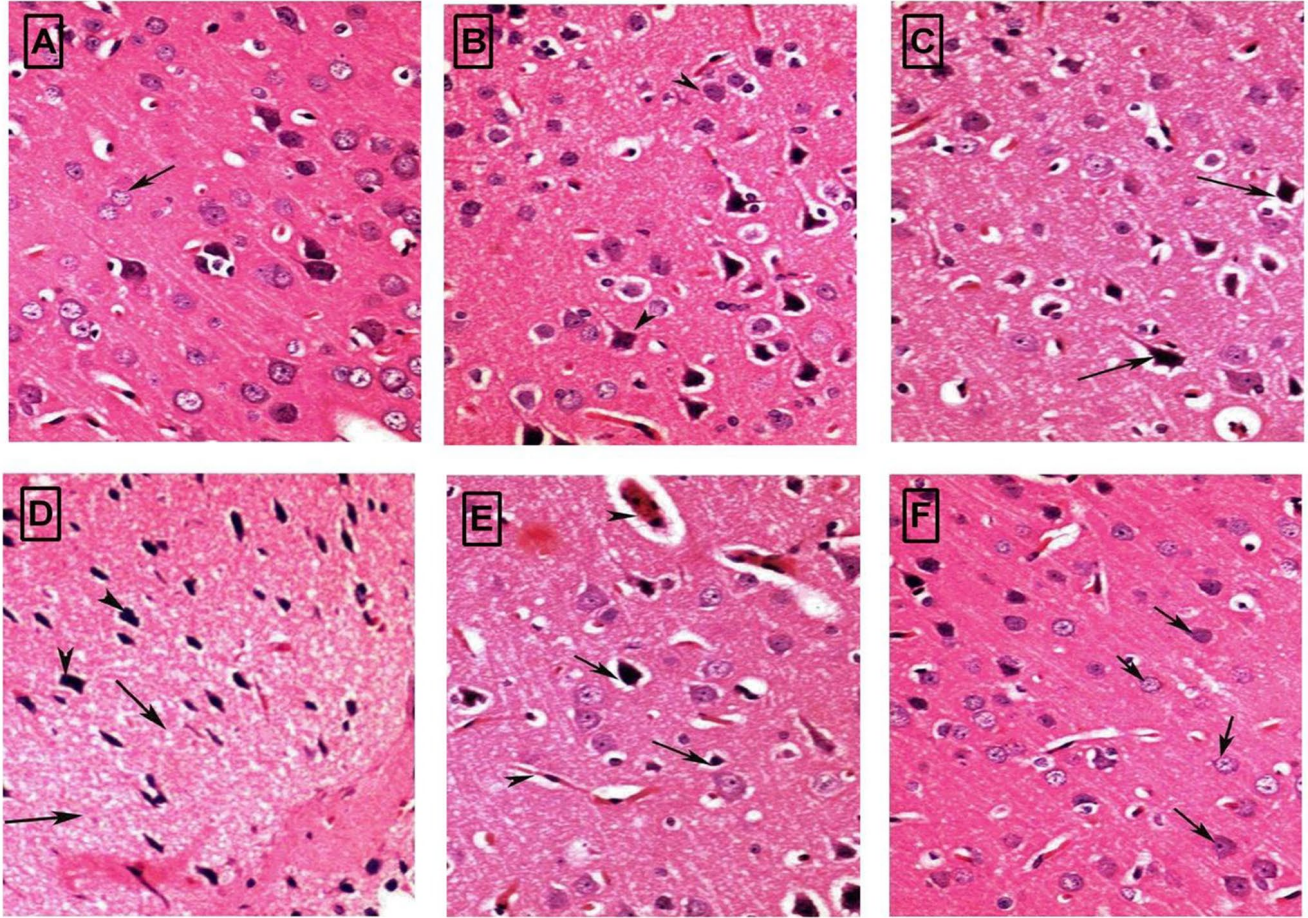
Histological changes of (H & E) stained sections from the rat brain (Cerebral cortex) were obtained from (**A**) control group showing no histopathological changes; (**B**) high salt (HS) group showing necrosis of neurons and neuronophagia; (**C**) HSD + PAM showing mild improvement but apoptotic (head) nuclei seeing; (**D**) HSD + PBA group showing improvement with mild cytoplasmic vacuolation (**E**) HSD + TAM group showing congestion of cerebral blood vessel, neuronal edema, necrosis of neurons and neuronophagia and (**F**) HSD + TBA group showing pronounced improvement of neurocytes with normal neurons (H & E × 400)

Cerebral cortex sections from control rats revealed the normal histological structure of the brain (Fig. 7A). Cerebral cortex sections from the HSD group indicated that high salt diet treatment showed degeneration of most Purkinje neurons, ill-defined chromatin, and faintly stained cytoplasm without clear Nissel's granules (Fig. 7B). Rats treated with amlodipine parallel to high salt diet showed mild improvement in Purkinje neurons (Fig. 7C). While rats treated with barley parallel to HSD treated group showed nearly restoration of the Purkinje cells to normal (Fig. 7D). The treatment with amlodipine for a month after stopping the high salt diet showed mild restoration Purkinje neurons to normal (Fig. 7E). Treatment with barley for a month after stopping the high salt diet an improvement in restoring the Purkinje layer to normal (Fig. 7F).

## Discussion

The notable finding of this study was that both prophylactic and therapeutic barley administration mitigated high salt-induced hypertensive responses by attenuating the oxidative and inflammatory responses and restoring the balance between excitatory and inhibitory neurotransmitters in the cerebellum of hypertensive rats.

In agreement with earlier studies, the present investigation demonstrates that a high salt diet induced a significant elevation in systolic and diastolic blood pressure with an increase in the generation of ROS in the cerebellum of HSD-fed rats as indicated by a high level of MDA in addition to low GSH level (Waken et al. 2017; Grillo et al. 2019; Zhang et al. 2020; Du et al. 2021; Leal et al. 2022). Numerous experimental and clinical findings have demonstrated the tight connection between salt-induced hypertension and the excessive generation of reactive oxygen species (ROS), which is crucial in regulating sympathetic nerve activity and the development of hypertension (Gao et al. 2017; Yu et al. 2021). It was reported that high salt consumption increased the amount of [Na +] in cerebrospinal fluid (CSF), activated the brain's RAS, and increased the synthesis of angiotensin II (Ang II) in rats (Huang et al. 2012). The binding of Ang II to the angiotensin II type-1 receptor (AT1R) contributes to blood pressure regulation (Yu et al. 2013). A previous study demonstrated that the elevated AT1R led to ROS buildup such as superoxide radical, hydroxyl ion, and hydrogen peroxide in the PVN, which might be involved in the pathogenesis of hypertension by causing sympathoexcitation as well as the overactivity of pre-autonomic PVN neurons (Zhang et al. 2020). Furthermore, PVN infusion of AT1R blocker decreased ROS generation, sympathetic nerve activity, and blood pressure (Yu et al. 2013). Additionally, A growing body of evidence demonstrated that nicotinamide-adenine dinucleotide phosphate [NADP(H)] oxidase-derived ROS is elevated in the different types of hypertension (Su et al. 2017). Moreover, inhibition of the NADP(H) oxidase complex attenuates ANGII-induced increases in superoxide production (Rajagopalan et al. 1996).

Additionally, this research’s results revealed that the HSD reduced NOx production in the cerebellum (Zheng et al. 2019; Leal et al. 2022). It was hypothesized that reactive oxygen species could render NOx inactive when there is oxidative stress on the tissue (NO + O^2−^ → ONOO^−^). The radical ONOO^−^ is a highly potent oxidant and nitrosating agent. As a result, this reaction produces the poisonous molecule ONOO^−^ and decreases NO availability. Reduced NO also contributes to arterial hypertension because it plays a crucial function as a vasodilator (Vaziri and Rodríguez-Iturbe 2006; Leal et al. 2022).

The results of this study confirmed that the high-salt diet caused neuroinflammation manifested by the significant rise in the levels of TNF-α and IL-1β in the cerebellum, which is consistent with previous findings that revealed a significant elevation in IL-1, IL-6, and TNF-α tissue levels in the PVN of Ang II-induced hypertensive rats (Knoll et al. 2017; Yu et al. 2021; Bhusal et al. 2021). Intracerebroventricular and PVN injections of IL-1 β increased the blood pressure of normotensive Sprague–Dawley rats (Shi et al. 2010), and infusion of an IL-1 inhibitor into the PVN of salt-sensitive hypertension rats attenuated hypertensive responses by reinstating the balance between pro- and anti-inflammatory cytokines and decreasing oxidative stress in the PVN (Qi et al. 2016). Similarly, in normotensive Sprague- Dawley rats, intracarotid infusion of TNF-α produced an increase in renal sympathetic nerve activity, BP, and heart rate (Yu et al. 2019). Consistently, central blockade of TNF-α prevents dysregulation of brain RAS components and attenuates Ang II-induced hypertension (Sriramula et al. 2011). Furthermore, intracerebroventricular infusion of TACE, an enzyme that frees membrane-attached TNF-α, increased blood pressure and sympathetic nerve activity in normotensive (Yu et al. 2019). Previous studies suggest that the actions of IL-1β and TNF-α on systemic arterial hypertension development seem to be focused on the central nervous system, influencing sympathetic drive by the activation of perivascular macrophages and increase of type 2 cyclooxygenase (COX- 2) expression/activity thus leading to increased production of prostaglandin E2 (PGE2) (Yu et al. 2010). Increased secretion of PGE2 by perivascular macrophages is believed to act on neuronal pathways within the PVN to increase the sympathetic drive to cardiovascular organs such as the heart and vasculature (Yu et al. 2010). IL-1β can also operate on the endothelial cells of fenestrated capillaries in the brain to cause sickness responses dependent on intact IL-1β signaling in blood vessels (Knoll et al. 2017).

Increasing evidence demonstrates that hypertension is associated with increased levels of excitatory neurotransmitters and decreased levels of inhibitory neurotransmitters in the PVN (Zhang et al. 2020; Du, et al. 2021; Leal et al. 2022). Consistent with previous studies, the results of the current research demonstrate that a high-salt diet caused a significant increase in dopamine, norepinephrine, and aspartate and a significant decrease in serotonin and GABA, supporting the hypothesis that high-salt diet-induced hypertension may result from the imbalance between the excitatory and inhibitory neurotransmitters (Gao et al. 2017; Yu et al. 2021). HSD can modulate the neuronal excitation of the rostral ventrolateral medulla and the firing activity of blood pressure-related neurons (Gao et al. 2017). It was suggested that HSD might increase sympathoexcitation and blood pressure by triggering the overproduction of ROS. The overproduction of hydroxyl and superoxide anions lowers the monoamine levels in the brain (Fedoce et al. 2018). Additionally, the results of the present study revealed that the AChE activity was significantly decreased in the cerebellum of HSD-fed rats. Previous studies showed that AChE activity was significantly and negatively correlated with the levels of TBARS and protein carbonyls, denoting that oxidative stress might contribute to the decreased AChE activity (Méndez-Garrido et al. 2014; Liu et al. 2017). Moreover, data from the present study provide evidence that brain cell energy was diminished in the cerebellum of high salt-fed rats. These results are consistent with a previous study that reported that HS intake decreased ATP production and impaired mitochondrial biosynthesis and bioenergetics (Jiang et al. 2018). HSD-induced mitochondrial damage leads to severe ATP depletion that induces loss of mitochondrial membrane potential (Gottlieb et al. 2003; Honda et al. 2005). Loss of mitochondrial potential has been shown to be a key event in the demise of neuronal monoamine (Honda et al. 2005).

The *Hordeum vulgare* (barley) treatment in the present study exerts antihypertensive activity against high salt-induced hypertension as it decreased the systolic and diastolic blood pressure, attenuated oxidative stress, decreased the levels of proinflammatory cytokines, and restored the disturbance in neurotransmitters in the brain. Barley is rich in several health-boosting components, such as β-glucans and tocols (Idehen et al. 2017). Moreover, barley has many phenolic compounds, such as proanthocyanidins, quinines, flavonols, chalcones, derivatives of benzoic and cinnamic acids, and flavones, flavanones, and amino phenolic compounds (Huang et al. 2020). Most of the antihypertensive activity attributed to barley extract may be related to these compounds that could exert their antihypertensive activity through different pathways (Gul et al. 2014; Ra et al. 2020). In this study, the use of ethanolic barley extract as prophylactic agent against cerebellum damage in salt induced hypertensive rats exhibited a marked improvement effects against oxidative stress, inflammation, reduced cell energy and disturbed neurotransmitter balance. Furthermore, in the therapeutic group, the same outcomes were nearly reached. However, it is worth mentioning that prophylactic treatment was associated with expressive shifts in most the study parameters.

The findings of the present investigation show that barley treatment attenuated the oxidative stress as indicated by the increase in the GSH and decrease in the MDA levels in the cerebellum of HSD-fed rats. Extensive evidence shows that barley administration decreases oxidative stress and enhances antioxidant activity in rats (Gul et al. 2014; Ra et al. 2020). This antioxidant activity of barley could be attributed to various barley constituents, including important antioxidants such as vitamin E, phytic acid, tocotrienols, and various phenolic acids (Idehen et al. 2017; Zeng et al. 2020). These compounds are potent free radical scavengers due to their ability to absorb and neutralize oxygen radicals. Additionally, zinc, which is found in barley in significant amounts (Gul et al. 2014), might protect against oxidative stress by stabilizing membranes by inhibiting the NADPH oxidase and stimulating the synthesis of metallothioneins, which reduce the levels of hydroxyl radicals and sequestering ROS (Marreiro et al. 2017). Moreover, Gul et al. (2014) showed that barley strengthens the antioxidant defense system and inhibits lipoxygenase and cyclooxygenase pathways of arachidonic metabolism.

Furthermore, barley administration in this study exerts anti-inflammatory effects as it reduced the levels of cerebral TNF-α and IL-1β in rats fed HSD. These findings support previous research that saponarin, the major component of barley seeds methanol extract, inhibited inflammation via NF-κB suppression (Seo et al. 2014). Similarly, Yang et al. (2021) reported that barley seeds extract or isolated lutonarin suppressed the lipopolysaccharide-induced upregulation of proinflammatory cytokines (IL)-6 and TNF-α and the inflammatory enzyme COX-2 and inducible nitric oxide synthase (iNOS). Additionally, barley treatment in the present study increased the bioavailability of the vasodilator nitric oxide. The antioxidant activity of barley extract could explain this increase in NOx concentration. Besides, arginine has been reported to be found in significant amounts in barley (Zeng et al. 2020). Thus, barley provides sufficient precursors for NOx synthesis and significantly increases NOx concentration (Ischiropoulos et al. 1996).

Moreover, barley treatment in the present study caused a restoration of the observed disturbances in the neurotransmitter levels in the cerebellum of high salt-fed rats. Barley is rich in tryptophan, tyrosine, and phenylalanine (Fernstrom and Fernstrom 2007; Jaeger et al. 2021). Aromatic amino acids in the brain function as precursors for the monoamine neurotransmitters serotonin (substrate tryptophan) and the catecholamines [dopamine, norepinephrine, epinephrine; substrate tyrosine (Tyr)] (Badrasawi et al. 2013). Thus, barley could regulate the synthesis of 5-HT, DA, and NE through the conversion of tryptophan to 5-hydroxytryptophan (5-HTP) to 5-HT and hydrolysis of phenylalanine to generate tyrosine that ultimately produces DA and NE (Badrasawi et al. 2013). Additionally, barley is rich in folic acid (Zeng et al. 2020), which is involved in the synthesis of monoamine neurotransmitters and modulates serotonergic, dopaminergic, and noradrenergic systems by acting as a cofactor for enzymes that convert tryptophan to 5-HT and enzymes that convert tyrosine to noradrenaline (Zhou et al. 2020).

Amlodipine, a widely used antihypertensive drug, relaxes smooth muscle in the heart and blood vessels by decreasing the influx of calcium ions through voltage-sensitive calcium channels (Rami and Krieglstein 1994). Calcium ions are the cause that lead to damage of the tissues in the heart and other organs, that results in stroke. Amlodipine was prescribed 75,811,947 times in the United States in 2018, making it the fifth mostly prescribed drug (Lee et al. 2021). Thus, in this study we aimed to compare the efficacy of barley ethanolic extract to mitigate the high salt induced-cerebellum damage in the hypertensive rats to this widely used antihypertensive drug. The results of the present study showed that amlodipine treatments decreased systolic and diastolic blood pressure in the rats fed high salt diet. These antihypertensive effects might be related to the increased level of the vasodilating NO which observed in the HSD fed rats treated with amlodipine. However, compared to rats treated with amlodipine parallel to HSD (HSD + PAM group), both prophylactic and therapeutic barely treatments displayed more expressive modification on reducing oxidative stress, inflammation, and energy deficit, as well as restoring neurotransmitters disturbance. These results indicate that the positive effects of barely extract are independent from reducing blood pressure and it could be mediated by antioxidative and anti-inflammatory effects.

## Conclusion

Data from this study indicate that the administration of seed extract of *Hordeum vulgare* *L.* attenuates high salt-induced cerebral injury and hypertensive responses. The underlying mechanism could be due to its antioxidant, anti-inflammatory, and vasodilating effects and its ability to reestablish the balance between excitatory and inhibitory neurotransmitters.

## Data Availability

The datasets generated during and/or analyzed during the current study are available from the corresponding author upon reasonable request.

## Abbreviations

5-HT: 5-Hydroxytryptamine
AchE: Acetylcholinesterase
ASP: Aspartic acid
DA: Dopamine
GABA: Gamma-aminobutyric acid
HSD: High salt group
IL-1 ß: Interleukin-1ß
MDA: Malondialdehyde
NOx: Nitric oxide
NE: Norepinephrine
PAM: Prophylactic amlodipine
PBA: Prophylactic barley
ROS: Reactive oxygen species
GSH: Reduced glutathione
TAM: Therapeutic amlodipine
TBM: Therapeutic barley
TNF- α: Tumor necrosis factor-α

## References

[CR1] Badrasawi MM, Shahar S, AbdManaf Z, Haron H (2013). Effect of Talbinah food consumption on depressive symptoms among elderly individuals in long term care facilities, randomized clinical trial. Clin Interv Aging.

[CR2] Bhusal A, Rahman MH, Suk K (2021). Hypothalamic inflammation in metabolic disorders and aging. Cell Mol Life Sci.

[CR3] Biod T, Sirota B, Watson A (1948). Practical clinical biochemistry.

[CR4] Borrelli S, Provenzano M, Gagliardi I, Michael A, Liberti ME, De Nicola L, Conte G, Garofalo C, Andreucci M (2020). Sodium intake and chronic kidney disease. Int J Mol Sci.

[CR5] Darwish IE, Maklad HM, Diab IH (2013). Behavioral and neuronal biochemical possible effects in experimental induced chronic mild stress in male albino rats under the effect of oral barley administration in comparison to venlafaxine. Int J Physiol Pathophysiol Pharmacol.

[CR6] Drury RB, Wallington EA (1980). Carleton’s histological technique.

[CR7] Du X, Yu L, Ling S, Xie J, Chen W (2021). High-salt diet impairs the neurons plasticity and the neurotransmitters-related biological processes. Nutrients.

[CR8] Ellman GL, Courtney KD, Andres V, Feather-Stone RM (1961). A new and rapid colorimetric determination of acetylcholinesterase activity. Biochem Pharmacol.

[CR9] Fedoce ADG, Ferreira F, Bota RG, Bonet-Costa V, Sun PY, Davies KJA (2018). The role of oxidative stress in anxiety disorder: cause or consequence?. Free Radical Res.

[CR10] Fernstrom JD, Fernstrom MH (2007). Tyrosine, phenylalanine, and catecholamine synthesis and function in the brain. J Nutr.

[CR11] Gao HL, Yu XJ, Liu KL, Shi XL, Qi J, Chen YM, Zhang Y, Bai J, Yi QY, Feng ZP, Chen WS, Cui W, Liu JJ, Zhu GQ, Kang YM (2017). PVN Blockade of p44/42 MAPK pathway attenuates salt-induced hypertension through modulating neurotransmitters and attenuating oxidative stress. Sci Rep.

[CR12] Gorun V, Proinov I, Baltescu V, Balaban G, Barzu O (1978). Modified Ellman procedure for assay of cholinesterases in crude enzymatic preparations. Anal Biochem.

[CR13] Gottlieb E, Armour SM, Harris MH, Thompson CB (2003). Mitochondrial membrane potential regulates matrix co, configuration and cytochrome c release during apoptosis. Cell Death Differ.

[CR14] Grillo A, Salvi L, Coruzzi P, Salvi P, Parati G (2019). Sodium intake and hypertension. Nutrients.

[CR15] Gul S, Ahmed S, Kifli N (2014). Multiple pathways are responsible for anti-inflammatory and cardiovascular activities of Hordeum vulgare L. J Transl Med.

[CR16] Heinrikson RL, Meredith SC (1984). Amino acid analysis by reverse-phase high-performance liquid chromatography: precolumn derivatization with phenylisothiocyanate. Anal Biochem.

[CR17] Honda HM, Korge P, Weiss JN (2005). Mitochondria and ischemia/reperfusion injury. Ann N Y Acad Sci.

[CR18] Huang BS, White RA, Bi L, Leenen FH (2012). Central infusion of aliskiren prevents sympathetic hyperactivity and hypertension in Dahl salt-sensitive rats on high salt intake. Am J Physiol Regul Integr Comp Physiol.

[CR19] Huang H, Gao X, Li Y, Tian P, Nima Y, Laba Z, Ci Z, Wei X, Qu J, Guan W, Liao W (2020). Content analysis of vitamins, dietary fibers and amino acids in a wide collection of barley (Hordeum vulgare L.) from Tibet, China. Bioinformation.

[CR20] Idehen E, Tang Y, Sang S (2017). Bioactive phytochemicals in barley. J Food Drug Anal.

[CR21] Ischiropoulos H, Beers MF, Ohnishi ST, Fisher D, Garner SE, Thom SR (1996). Nitric oxide production and perivascular nitration in brain after carbon monoxide poisoning in the rat. J Clin Investig.

[CR22] Jaeger A, Zannini E, Sahin AW, Arendt EK (2021). Barley protein properties, extraction and applications, with a focus on brewers' spent grain protein. Foods (Basel, Switzerland).

[CR23] Jayatilleke E, Shaw S (1993). A high-performance liquid chromatographic assay for reduced and oxidized glutathione in biological samples. Anal Biochem.

[CR24] Jiang E, Chapp AD, Fan Y, Larson RA, Hahka T, Huber MJ, Yan J, Chen QH, Shan Z (2018). Expression of proinflammatory cytokines is upregulated in the hypothalamic paraventricular nucleus of Dahl salt-sensitive hypertensive rats. Front Physiol.

[CR25] Karatepe M (2004). Simultaneous determination of ascorbic acid and free malondialdehyde in human serum by HPLC-UV. LC-GC N Am.

[CR26] Kim H, Lee SH, Jung J, Hong S, Lim S (2022). Pharmacokinetic drug interaction between amlodipine and Tadalafil: an open-label, randomized, multiple-dose crossover study in healthy male volunteers. Drug Des Dev Ther.

[CR27] Knoll J, Krasnow SM, Marks DL (2017). Interleukin-1β signaling in fenestrated capillaries is sufficient to trigger sickness responses in mice. J Neuroinflammation.

[CR28] Leal PEPT, da Silva AA, Rocha-Gomes A, Riul TR, Cunha RA, Reichetzeder C, Villela DC (2022). High-salt diet in the pre- and postweaning periods leads to amygdala oxidative stress and changes in locomotion and anxiety-like behaviors of male Wistar rats. Front Behav Neurosci.

[CR29] Lee S, Jo C, Choi Y, Lee K (2021) Effect of co-administration of curcumin with amlodipine in hypertension. Nutrients 13(8). 10.3390/nu13082797 10.3390/nu13082797 PMC8399053 34444956

[CR30] Liu H, Wu J, Yao JY, Wang H, Li ST (2017). The role of oxidative stress in decreased acetylcholinesterase activity at the neuromuscular junction of the diaphragm during sepsis. Oxid Med Cell Long.

[CR31] Marreiro DD, Cruz KJ, Morais JB, Beserra JB, Severo JS, de Oliveira AR (2017). Zinc and oxidative stress: current mechanisms. Antioxidants (Basel, Switzerland).

[CR32] Marsh WH, Fingerhut B, Miller H (1965). Automated and manual direct methods for the determination of blood urea. Clin Chem.

[CR33] Mawatari E, Hongo M, Sakai A, Terasawa F, Takahashi M, Yazaki Y, Kinoshita O, Ikeda U (2007). amlodipine prevents monocrotaline-induced pulmonary arterial hypertension and prolongs survival in rats independent of blood pressure lowering. Clin Exp Pharmacol Physiol.

[CR34] Méndez-Garrido A, Hernández-Rodríguez M, Zamorano-Ulloa R, Correa-Basurto J, Mendieta-Wejebe JE, Ramírez-Rosales D, Rosales-Hernández MC (2014). In vitro effect of H2O2, some transition metals and hydroxyl radical produced via fenton and fenton-like reactions, on the catalytic activity of AChE and the hydrolysis of ACh. Neurochem Res.

[CR35] OldeEngberink RHG, van den Hoek TC, van Noordenne ND, van den Born BH, Peters-Sengers H, Vogt L (2017). Use of a single baseline versus multiyear 24-hour urine collection for estimation of long-term sodium intake and associated cardiovascular and renal risk. Circulation.

[CR36] Pagel P, Blome J, Wolf HU (2000). High-performance liquid chromatographic separation and measurement of various biogenic compounds possibly involved in the pathomechanism of Parkinson's disease. J Chromatogr B Biomed Sci Appl.

[CR37] Papadoyannis LN, Samanidou VF, Nitsos ChC (1999). Simultaneous determination of nitrite and nitrate in drinking water and human serum by high performance anion-exchange chromatography and UV detection. J Liq Chrom Rel Technol.

[CR38] Qi J, Zhao XF, Yu XJ, Yi QY, Shi XL, Tan H, Fan XY, Gao HL, Yue LY, Feng ZP, Kang YM (2016). Targeting Interleukin-1 beta to suppress sympathoexcitation in hypothalamic paraventricular nucleus in Dahl salt-sensitive hypertensive rats. Cardiovasc Toxicol.

[CR39] Qin J, He Z, Wu L, Wang W, Lin Q, Lin Y, Zheng L (2021). Prevalence of mild cognitive impairment in patients with hypertension: a systematic review and meta-analysis. Hypertens Res: Off J Jpn Soc Hypertens.

[CR40] Ra J, Woo S, Jin H, Lee MJ, Kim HY, Ham H, Chung I, Seo WD (2020). Evaluation of antihypertensive polyphenols of barley (Hordeum vulgare L.) seedlings via their effects on angiotensin-converting enzyme (ACE) inhibition. Appl Biol Chem.

[CR41] Rajagopalan S, Kurz S, Münzel T, Tarpey M, Freeman BA, Griendling KK, Harrison DG (1996). Angiotensin II-mediated hypertension in the rat increases vascular superoxide production via membrane NADH/NADPH oxidase activation. Contribution to alterations of vasomotor tone. J Clin Investig.

[CR42] Rami A, Krieglstein J (1994). Neuronal protective effects of calcium antagonists in cerebral ischemia. Life Sci.

[CR43] Reeves PG, Nielsen FH, Fahey GC (1993). AIN-93 purified diets for laboratory rodents: final report of the American Institute of Nutrition ad hoc writing committee on the reformulation of the AIN-76A rodent diet. J Nutr.

[CR44] Seo KH, Park MJ, Ra JE, Han SI, Nam MH, Kim JH, Lee JH, Seo WD (2014). Saponarin from barley sprouts inhibits NF-κB and MAPK on LPS-induced RAW 264.7 cells. Food Funct.

[CR45] Shi P, Raizada MK, Sumners C (2010). Brain cytokines as neuromodulators in cardiovascular control. Clin Exp Pharmacol Physiol.

[CR46] Sriramula S, Cardinale JP, Lazartigues E, Francis J (2011). ACE2 overexpression in the paraventricular nucleus attenuates angiotensin II-induced hypertension. Cardiovasc Res.

[CR47] Su Q, Huo CJ, Li HB, Liu KL, Li X, Yang Q, Song XA, Chen WS, Cui W, Zhu GQ, Shi XL, Liu JJ, Kang YM (2017). Renin-angiotensin system acting on reactive oxygen species in paraventricular nucleus induces sympathetic activation via AT1R/PKCγ/Rac1 pathway in salt-induced hypertension. Sci Rep.

[CR48] Teerlink T, Hennekes M, Bussemaker J, Groeneveld J (1993). Simultaneous determination of creatine compounds and adenine nucleotides in myocardial tissue by high-performance liquid chromatography. Anal Biochem.

[CR49] Vaziri ND, Rodríguez-Iturbe B (2006). Mechanisms of disease: oxidative stress and inflammation in the pathogenesis of hypertension. Nat Clin Pract Nephrol.

[CR50] Waken RJ, de Las FL, Rao DC (2017). A review of the genetics of hypertension with a focus on gene-environment interactions. Curr Hypertens Rep.

[CR51] Walkowska A, Kuczeriszka M, Sadowski J, Olszyñski KH, Dobrowolski L, Červenka L, Hammock BD, Kompanowska-Jezierska E (2015). High salt intake increases blood pressure in normal rats: putative role of 20-HETE and no evidence on changes in renal vascular reactivity. Kidney Blood Press Res.

[CR52] Yang JY, Woo SY, Lee MJ, Kim HY, Lee JH, Kim SH, Seo WD (2021). Lutonarin from Barley seedlings inhibits the lipopolysacchride-stimulated inflammatory response of RAW 264.7 macrophages by suppressing nuclear Factor-κB signaling. Molecules (Basel, Switzerland).

[CR53] Yu Y, Zhang ZH, Wei SG, Serrats J, Weiss RM, Felder RB (2010). Brain perivascular macrophages and the sympathetic response to inflammation in rats after myocardial infarction. Hypertension (Dallas, Tex. : 1979).

[CR54] Yu XJ, Suo YP, Qi J, Yang Q, Li HH, Zhang DM, Yi QY, Zhang J, Zhu GQ, Zhu Z, Kang YM (2013). Interaction between AT1 receptor and NF-κB in hypothalamic paraventricular nucleus contributes to oxidative stress and sympathoexcitation by modulating neurotransmitters in heart failure. Cardiovasc Toxicol.

[CR55] Yu Y, Cao Y, Bell B, Chen X, Weiss RM, Felder RB, Wei SG (2019). Brain TACE (Tumor Necrosis Factor-α-Converting Enzyme) contributes to sympathetic excitation in heart failure rats. Hypertension (Dallas, Tex. : 1979).

[CR56] Yu X, Xiao T, Liu X, Li Y, Qi J, Zhang N, Fu L, Liu K, Li Y, Kang Y (2021) Effects of Nrf1 in hypothalamic paraventricular nucleus on regulating the blood pressure during hypertension. Front Neurosci 15. 10.3389/fnins.2021.805070 10.3389/fnins.2021.805070 PMC8685333 34938159

[CR57] Zeng Y, Pu X, Du J, Yang X, Li X, Mandal MSN, Yang T, Yang J (2020). Molecular mechanism of functional ingredients in Barley to combat human chronic diseases. Oxid Med Cell Longev.

[CR58] Zhang T, Wang D, Li X, Jiang Y, Wang C, Zhang Y, Kong Q, Tian C, Dai Y, Zhao W, Jiang M, Chang Y, Wang G (2020). Excess salt intake promotes M1 microglia polarization via a p38/MAPK/AR-dependent pathway after cerebral ischemia in mice. Int Immunopharmacol.

[CR59] Zheng X, Li X, Chen M, Yang P, Zhao X, Zeng L, OuYang Y, Yang Z, Tian Z (2019). The protective role of hawthorn fruit extract against high salt-induced hypertension in Dahl salt-sensitive rats: impact on oxidative stress and metabolic patterns. Food Funct.

[CR60] Zhou Y, Cong Y, Liu H (2020). Folic acid ameliorates depression-like behaviour in a rat model of chronic unpredictable mild stress. BMC Neurosci.

